# Fetal Safety of Intravenous Ferric Carboxymaltose in Pregnancy: A Cardiotocography Study from a Tertiary Care Hospital in Italy

**DOI:** 10.3390/hematolrep18010007

**Published:** 2026-01-05

**Authors:** Francesca Polese, Chiara Pesce, Giulia De Fusco, Gianni Tidore, Enza Coluccia, Raffaele Battista, Gianluca Gessoni

**Affiliations:** 1 Transfusion Medicine Unit, Dell’Angelo General Hospital, Via Paccagnella 11, Mestre, 30172 Venice, Italy; 2 Obstetrics and Gynecology Unit, Dell’Angelo General Hospital, Mestre, 30172 Venice, Italy; 3 Transfusion Medicine Department, Dell’Angelo General Hospital, Via Paccagnella 11, 30172 Mestre, Italy

**Keywords:** cardiotocography, ferric carbossimaltose, iron deficient anemia, pregnancy

## Abstract

**Background:** Iron-deficient anemia (IDA) in pregnant women is a significant health issue globally. Oral iron supplementation is the primary treatment for IDA during pregnancy. For women who do not respond to or cannot tolerate oral iron treatment, intravenous (IV) iron preparations may offer a viable therapeutic option in the third trimester of pregnancy. Ferric carboxymaltose (FCM; Ferinject^®^) is an IV iron preparation that allows rapid administration of high single doses of iron with a favorable safety profile. This study evaluated the potential impact of FCM therapy on fetal well-being by recording cardiotocography (CTG) before, during, and after iron infusions. **Materials and Methods:** We examined 105 women with IDA in the third trimester of pregnancy. During the initial evaluation, each patient was assessed for complete blood count, iron metabolism, B12, folates, hemoglobinopathies, CRP, kidney and liver function, and glucose levels. Each subject received intravenous ferric carboxymaltose (FCM), 500 mg. The study focused on the maternal and fetal safety of FCM infusion. The primary endpoint for maternal safety was the observation of adverse effects of iron infusion. For fetal safety, the primary endpoint was the assessment of CTG. **Results:** We considered 105 women, comprising 101 singleton and 4 twin pregnancies. The median hemoglobin (Hb) at initial observation was 95 g/L and 117 g/L post-therapy. Regarding maternal safety, side effects were observed during or after FCM infusion in four subjects; three cases involved local symptoms, while one case included nausea and skin rash. Concerning fetal safety, 100% of the cardiotocography records were deemed “normal” using the Dawes–Redman criteria. **Conclusions:** In conclusion, FCM proved effective in treating anemia in this clinically complex population of pregnant women in the third trimester and appeared safe in this cohort, though larger prospective studies are warranted.

## 1. Background

Approximately 1.24 billion people worldwide are affected by iron deficiency anemia (IDA), especially women, low–middle social demographic groups, as well as populations of Asia and sub-Saharan Africa [1]. Anemia in pregnancy is thought to affect 32 million women worldwide [2,3]. A large, UK cohort study reported that 46% of women were anemic at some point during pregnancy [4]. Iron deficiency (ID) is the most common cause of maternal anemia. Other causes include haemoglobinopathies, deficiencies of folates and/or B12, viral infection such as HIV, or hookworm infestation [5]. The association between anemia and poor maternal, fetal, and neonatal outcomes such as preterm labor, growth restriction, and increased mortality is well established [6,7].

Oral iron supplements are the first-line treatment for IDA during pregnancy but can cause gastrointestinal side effects, such as nausea and diarrhea, that impact the adherence to and, ultimately, the efficacy of the iron treatment [8,9]. For those women who do not respond to oral iron treatment, intravenous (IV) iron preparations may provide a better option, especially for women with severe IDA diagnosed later in pregnancy [10,11]. IV iron preparations can deliver larger doses quickly without the risk of gastrointestinal side effects. However, some currently available IV iron preparations require multiple doses and long infusion times; moreover, they are not free from side effects on the mother and the fetus. Ferric carboxymaltose (FCM; Ferinject^®^) is a dextran-free IV iron preparation that allows rapid administration of high single doses of iron (up to 500 mg iron in around 30 min) and, in the general population, appears to be burdened by a lower incidence of side effects than other iron-based preparations [12,13]. FCM is widely used in the treatment of IDA during pregnancy [14,15]. However, to date, no adequate data are available to evaluate the tolerability profile of FCM on the fetus. So, our study provides new evidence using cardiotocographic (CTG) monitoring.

In this paper, we evaluated the possible impact of FCM therapy on fetal well-being, assessed by recording the fetal heartbeat through CTG tracing, before, during, and after the infusion.

## 2. Materials and Methods

Study design: This is a single-center retrospective study performed in a large tertiary care urban hospital in the Veneto Region (north-east Italy). The study was conducted in accordance with the Helsinki Declaration. The study was authorized by the Clinical Research Ethics Committee of the Province of Venice (154A/CESC, 12 May 2022). Each patient provided written informed consent for intravenous ferric carboxymaltose (FCM) infusion and cardiotocography (CTG) registration. Furthermore, each patient provided a written release for the use of their data for scientific purposes. All enrolled subjects provided written informed consent to the Laboratory, and clinical data were obtained from routine clinical registration.

Patients: Pregnant women, aged over 18 years, in the third trimester with iron deficiency (ID), defined as serum ferritin < 15 μg/mL and/or transferrin saturation (TSAT) < 20%, who had ineffective or not tolerated oral iron administration, were referred to our clinics. At initial evaluation, each patient underwent a complete blood count, serum iron, transferrin, transferrin saturation, ferritin, B12 vitamin, folates, screening for hemoglobinopathies, C-reactive protein, kidney function, liver function, phosphates, and glucose metabolism assessment. A thorough clinical history was also collected, particularly focusing on previous obstetric history, allergies or intolerances, presence of concomitant pathologies. Patients referred to our hub facility were selected based on clinical complexity, severity of anemia, and management challenges. The patient cohort exhibited a high incidence of concomitant pathologies, including diabetes (13.3%), hypothyroidism (11.4%), hypertension (7.6%), and recurrent miscarriages (3.8%). One patient had undergone bariatric surgery, known to interfere with the absorption of iron, vitamin B12, and folates, posing a significant risk factor for anemia during pregnancy [16]. Additionally, 17.1% of patients presented disorders of hemoglobin synthesis, including heterozygous beta thalassemia, hemoglobin E, and hemoglobin S, correlating with the geographical diversity of the patients’ origins [17,18]. Hemoglobin concentrations were not significantly different between women with and without hemoglobinopathies.

Iron administration: Each subject received intravenous ferric carboxymaltose (FCM), 500 mg, in 250 mL of normal saline, infused in 30 min; FCM was always administered in a 50 mg/mL solution in a 10 mL vial. The dose was repeated if necessary in patients with an unsatisfactory response to therapy. After each FCM infusion, patients were observed for at least 20 min.

Cardiotocography: CTG acquisition was performed using Philips Avalon FM20 or Philips Avalon FM30 equipment (Philips Hospital Supplies and Equioemen, Itlia, Milan, Italy) with portable wired sensors that transmit signals to a remote fetal monitor (telemetry). This method allows the mother to move freely during signal acquisition. For the horizontal scale for CTG registration, a 1 cm/min was selected. For cardiotocography, we recorded 10 min of CTG prior to therapy, then continuous monitoring during FCM infusion lasting about 30 min, and at least 20 min after the administration’s conclusion. At the end of the CTG registration, a “computerised” CTG analysis was performed, incorporating the Dawes–Redman criteria, which must be fulfilled in order to terminate fetal surveillance, are analyzed [19,20]. Signal loss (must be less than 30%) represents the percentage of how long the CTG was unable to detect the fetal heartbeat and is considered a marker of the quality of the CTG trace [19,20]. Fetal heart rate (FHR) should be between 110 and 160 bpm [19,20]. Oscillations in the FHR, evaluated as the average bandwidth amplitude of the signal in one-minute registration, a bandwidth amplitude of 5–25 bpm is considered normal. In a normal CTG record, at least one heart rate acceleration over 10 bpm and/or over 20 bpm for a minimum of 15 s must be present; moreover, FHR deceleration, defined as a decrease of more than 10 bpm for more than 1 min, or of 20 bpm for more than 30 s, should be considered [19,20]. Short-term variability represents the beat-to-beat variability and is the most reliable prognostic parameter linked to fetal well-being. It must be greater than 4 ms [19,20]. High variability: the computer calculates the difference between the minimum and maximum of the “pulse interval” during each minute, obtaining a value called the minute range. If the latter exceeds 32 milliseconds (ms) for at least 5 out of 6 consecutive minutes, the system signals this event as the start of a high variability episode. At least 1 high variability episode must be present [21]. In our patient series, the MED number of high variability episodes was 10, and 100% of CTG records met the requirements. Low variability: Amplitude is less than 5 bpm for more than 50 min; in a normal CTG record, there should be no period of low variability [19,20]. Sinusoidal pattern is defined as a regular, smooth, undulating signal, resembling a sine wave, with an amplitude of 5–15 bpm, and a frequency of 3–5 cycles per minute, lasting more than 30 min, and coincides with absent accelerations and often is observed in association with severe fetal anemia [19,20]. Recording of fetal movement and recording of uterine contraction are parameters that do not affect the CTG record interpretation criteria, but give operators an additional key to interpreting the records [19,20].

Endpoints: This study was focused on the maternal and fetal safety of FCM infusion. The primary endpoint of maternal safety was the observation of side effects of iron infusion. The primary endpoint for fetal safety was the observation of abnormal CTG records. Regarding maternal safety, secondary endpoints were considered an increase in maternal Hb concentration, need for additional CMF infusion, and requirement for packed red cells (PRC) transfusion. Regarding fetal safety, secondary endpoints included preterm delivery and a five-minute APGAR score less than 7 [22,23].

Calculations and Statistical analysis: Statistical analysis was performed using the MedCalc Software v 11.1.0 (MedCalc Software Ltd., Ostend, Belgium). First, using the D’Agostino-Pearson test, data distribution was evaluated; having rejected the hypothesis of a “normal” distribution, a non-parametric statistical approach was employed. Categorical data are presented as numbers (percentages) and continuous data as median (MED) and interquartile range (IQR). For data comparison, the Kruskal–Wallis test we adopted, with an Alpha level defined as *p* < 0.05 considered statistically significant.

## 3. Results

**Figure 1:**
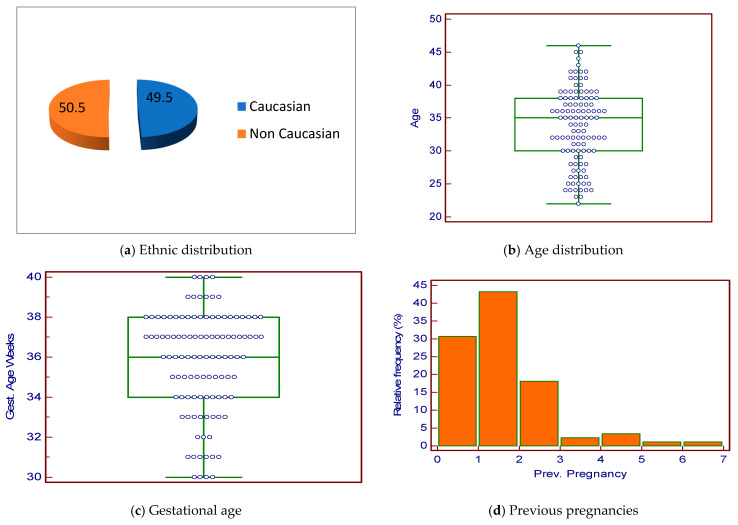
Patients’ personal data of 105 pregnant women.

In the 24 months between January 2023 and December 2024, 237 women were referred to our clinics for evaluation of anemia in pregnancy. Among patients with severe iron-deficiency anemia diagnosed later in pregnancy, those who did not respond to, were non-compliant with, or could not tolerate oral iron treatment, 105 patients were selected for treatment with ferric carboxymaltose (FCM). Of these patients, 52 (49.5%) were Caucasian (Figure 1a). The median age of these 105 patients was 34.5 years (IQR 8 years), all of whom were in the third trimester of pregnancy with a median gestational age of 36 weeks (IQR 3.5 weeks) (Figure 1b,c). Data regarding previous pregnancies are reported in Figure 1d. In this group, there were 101 (96.2%) singleton pregnancies and 4 (3.8%) twin pregnancies.

Clinical history of these patients revealed several co-morbidities: diabetes mellitus in 14 cases (13.3%), hypothyroidism in 12 (11.4%), arterial hypertension in 8 (7.6%), history of multiple abortions in 4 (3.8%), diseases affecting the CNS or thyroiditis in 3 (2.9%) each, tuberculosis in 2 (1.9%), and previous pulmonary thromboembolism, rheumatoid arthritis, severe asthma, sleeve gastrectomy, and previous tetralogy of Fallot in one case (0.9%) each. These data are reported in Table 1. Among the obstetric conditions highlighted in the current pregnancy, three cases of small-for-gestational-age fetuses and one case of placenta previa should be noted. The results of the drug history are reported in Table 2. Patients’ symptoms were reported in Table 3.

**Figure 2:**
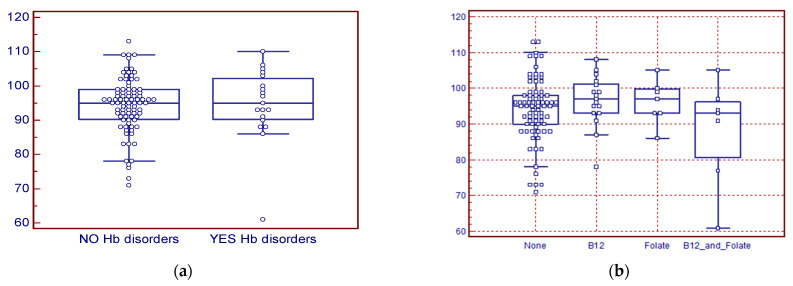

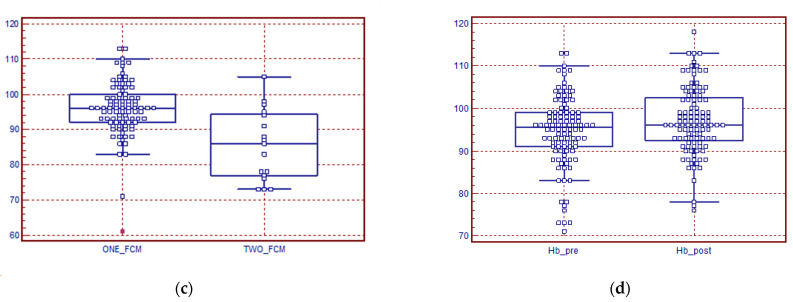
Distribution of Hemoglobin (Hb) concentration. (**a**) Hb concentration in women with and without hemoglobin disorders. (**b**) Hb concentration in women without and with B12 and folate deficiency. (**c**) Hb concentration in women who received one or two infusions of 500 mg of Ferric carboxymaltose (FCM). (**d**) Hb concentration before and after therapy with intravenous FCM.

Considering laboratory parameters, as reported in Table 4, the median hemoglobin (Hb) at first observation was 95 g/L (IQR 0.9 g/L), the mean corpuscular volume (MCV) was 78 fL (IQR 13 fL), median ferritin concentration was 8 ng/mL (IQR 11 ng/mL), and median transferrin saturation was 10% (IQR 11%). A total of 18 subjects presented an Hb disorder: 13 (12.4%) patients had heterozygous Beta thalassemia, 3 (2.9%) had homozygous HbE, and 2 (1.9%) had homozygous HbS. In subjects without hemoglobinopathies, the median Hb concentration was 96 g/L (IQR 8 g/L), while in subjects with hemoglobinopathies it was 95 g/L (IQR 11 g/L). This difference was not statistically significant (*p* = 0.99); for the distribution of Hb values in the two groups, see Figure 2a. Among this patient series, we observed 74 (70.5%) women with normal levels of vitamin B12 and folates; in this group, the median Hb concentration was 95 g/L (IQR 8 g/L); 17 (16.2%) had low levels of vitamin B12, with a median Hb concentration of 97 g/L (IQR 9 g/L); 7 (6.7%) had low levels of folates, with a median Hb concentration of 96 g/L (IQR 7 g/L); and 7 (6.9%) had deficits in both B12 and folates, with a median Hb concentration of 93 g/L (IQR 11 g/L). None of the hemoglobin concentration values differed significantly among the four groups; for the distribution of observed Hb values, see Figure 2b.

In the 105 women considered, 122 infusions of FCM were performed before delivery, as 17 (16.2%) patients required a second dose of FCM. Patients requiring two FCM infusions had a median Hb of 86 g/L (IQR 17 g/L), while those requiring only one infusion had a median Hb of 97 g/L (IQR 8 g/L); this difference was statistically significant (*p* = 0.002); for the distribution of observed Hb values, see Figure 2c. Four patients (3.8%) experienced side effects during or after FCM infusion, three of whom had local symptoms starting from the infusion site, and one case included nausea and skin rash. No serious side effects like hypotension, precordial pain, or syncope were observed. Additionally, due to severe symptomatic anemia, four patients received blood transfusions, with a total of seven units of packed red cells administered; no post-transfusion adverse events were observed. The median Hb concentration in transfused patients was 69 g/L (IQR 9 g/L), significantly lower (*p* < 0.001) than in non-transfused subjects (median 96 g/L, IQR 9 g/L). The median Hb concentration after therapy, measured at hospital admission before delivery, was 117 g/L (IQR 1.5 g/L), with an average increase in Hb concentration of 22 g/L. This difference was statistically significant (*p* < 0.001). See Figure 2d for the Hb values distribution before and after intravenous FCM.

Figure 2a shows the distribution of hemoglobin (g/L) values observed in women with and without disorders of hemoglobin synthesis. The observed difference was not statistically significant (*p* = 0.1). Figure 2b shows the distribution of hemoglobin (g/L) values observed in women with and without vitamin B12 and/or folate deficiency. The observed differences were not statistically significant (*p* = 0.4). Figure 2c shows the distribution of hemoglobin (g/L) values observed in women who received a single dose of FCM compared to women who received a second infusion. The observed difference was statistically significant (*p* = 0.002). Figure 2d shows the distribution of hemoglobin (g/L) values observed before and after intravenous FCM therapy. The observed difference was statistically significant (*p* < 0.001).

Considering the number of patients, FCM infusions performed, and the presence of four twin pregnancies, 128 cardiotocographies were conducted to monitor fetal health. As reported in Table 5, compliance of the cardiotocography records with the Dawes–Redman requirements [19,20] was evaluated, showing the following parameters: signal loss (%), with acceptance criteria < 30%, observed values median 4%, IQR 9%, CTG meeting acceptance criteria 100%. Fetal heart rate (bpm), acceptance criteria 110–160 bpm, observed values median 137 bpm, IQR 10 bpm, CTG meeting acceptance criteria 100%. Fetal heart rate acceleration between 10 and 20 bpm for at least 15 s, acceptance criteria > 1 episode, observed values median 3 episodes, IQR 1.8 episodes, CTG meeting acceptance criteria 100%. Fetal heart rate acceleration over 20 bpm for at least 15 s, acceptance criteria > 1 episode, observed values median 3 episodes, IQR 2.0 episodes, CTG meeting acceptance criteria 100%. Fetal heart rate decelerations, defined as a decrease in more than 10 bpm for more than 1 min, or of 20 bpm for more than 30 s, acceptance criteria < 1 episode, observed values median 0 episodes, IQR 1.0 episode, CTG meeting acceptance criteria 100%. There must be no decelerations greater than 20 bpm, observed values median 0, IQR 0, CTG meeting acceptance criteria 100%. Short-term variability (ms), acceptance criteria > 4 ms, observed values median 12.9 ms, IQR 5.9 ms, CTG meeting acceptance criteria 100%. High variability, with at least 1 episode present, observed values median 10 episodes, IQR 4 episodes, CTG meeting acceptance criteria 100%. Low variability, defined as variation in less than 5 bpm for 50 min, acceptance criteria “absence”, observed values median 0, IQR 0, CTG meeting acceptance criteria 100%. Sinusoidal pattern acceptance criteria “absence”, observed values median 0, IQR 0, CTG meeting acceptance criteria 100%. Fetal movement, median 5, IQR 11; uterine contractions, median 2, IQR 1. Therefore, 128/128 (100%) of the cardiotocography record results were “normal” according to the Dawes–Redman criteria [19,20].

No preterm deliveries were observed. The mean five-minute APGAR Score was 9.5 (2DS 0.5), and no newborn had an APGAR score of less than 7 at five minutes.

## 4. Discussion

This single-center retrospective study reviews the routine use of intravenous ferric carboxymaltose during the third trimester of pregnancy in a tertiary care hospital in the Veneto Region (north-east Italy). Typically, patients were sent to our facility because of a complex clinical situation, such as an iron deficiency anemia unresponsive to oral iron therapy or proximity to the expected date of delivery, requiring rapid anemia assessment and treatment. Upon initial observation, our testing protocol included a complete whole blood count, evaluation of iron metabolism, dosages of vitamin B12 and folates, assessments of anomalies in globin chains synthesis, liver and kidney function studies, and evaluation of inflammatory states.

A first notable aspect of our case study is the high percentage of non-Caucasian women, constituting 50.5% of the treated patients; a percentage almost five times greater compared to the 10.5% of foreign residents in the Veneto region as of 31 December 2023 [24]. Non-Caucasian immigrants often belong to disadvantaged socio-economic groups, often presenting cultural and linguistic barriers that make it difficult the access high standards of care. These subjects often experience repeated and close pregnancies that can accentuate deficiencies of vitamins or essential nutrients [25,26]. These considerations may help to explain the high incidence (31%) of vitamin B12 and/or folate deficiency in our series. Despite this, hemoglobin concentration did not differ significantly comparing women without and with vitamin B12 and/or folate deficiency. We believe, however, that the dosage of vitamin B12 and folates should always be performed in patients referred to specialized facilities to correctly classify anemia and plan any corrective treatments [27,28,29]. Particularly in cases such as ours, characterised by high complexity, with a high percentage of comorbidities and haemoglobinopathies [16,17,18].

Regarding maternal safety, FCM therapy was well tolerated with an overall low incidence of adverse reactions (3.8%), and only one (0.8%) required the suspension of the infusion and recourse to pharmacological therapy. Therefore, regarding this primary endpoint, we can conclude that the infusion of FCM in the third trimester of pregnancy is well tolerated with a negligible incidence of serious adverse effects; these results are in good accord with data from the literature [30,31]. Secondary endpoints concerning maternal safety include a rise in maternal Hb concentration, need for further FCM infusions, and packed red cell transfusion requirements. In this patient series, we observed a satisfactory increase in maternal Hb concentration, which went from a median value of 95 g/L to a median value of 117 g/L with a median increase of 22 g/L. We can therefore affirm, on the basis of the results obtained, that the infusion of FCM is able to rapidly and satisfactorily increase the concentration of hemoglobin in women in the third trimester of pregnancy [32,33]. It is noteworthy that three patients were diagnosed with small-for-gestational-age fetuses, a condition that has been associated with IDA in pregnancy [34]. In our series, we observed 17 patients (16.2%) with marked IDA that required further administration of FCM (500 mg). Although there are experiences in the literature that suggest the possibility of using up to 1000 mg of FCM in a single infusion, in our structure, we decided to maintain the standard dosage of 500 mg and repeat the therapy if necessary [35]. Four patients (3.8%) with severe, symptomatic anemia required PRC transfusions. It should be noted that three of these also presented a hemoglobinopathy (two HbS and one beta thalassemia) [36].

Regarding fetal safety, the primary endpoint was the absence of abnormal CTG records. To perform CTG registration, maternal lateral recumbent, half-sitting, and upright positions were adopted using Philips Avalon instrumentation with wired portable sensors that transmit signals to a remote fetal monitor (telemetry). This solution has the advantage of avoiding aorto-caval compression, allowing the mother to move freely during signal acquisition with improved comfort during the procedure [37]. As recommended, monitoring of twin pregnancies was performed using a continuous dual-channel monitors that allow simultaneous monitoring of both fetal heart rate [38]. Each recording lasted at least 60 min. At the end of registration, a computerized CTG analysis using the Dawes–Redman criteria was performed [19,20,39,40,41,42]. Signal loss must be less than 30% and is considered a marker of the quality of the CTG trace [39]. In our patient series, the MED signal loss was 4% and none was over 30%; therefore, all the tracks were compliant with the requirement. Fetal heart rate (FHR) should be between 110 and 160 bpm; all the tracks were compliant with the requirement [40]. Variability refers to the oscillations in the FHR, evaluated as the average bandwidth amplitude of the signal in one-minute registration; a bandwidth amplitude of 5–25 bpm is considered normal [41,42]. In our patient series, all CTG records meet the normality criteria for FHR variability reported above. Short-term variability indicates the micro-fluctuations in the heart rate between one beat and the next. It is the most reliable prognostic parameter linked to fetal well-being [21,42]. It must be greater than 4 ms. In our patient series, the MED short-term variability was 12.9 ms, and none was under 4 ms; therefore, all the tracks were compliant with the requirement. Considering the high variability parameter, at least one high variability episode must be present [21]. In our patient series, the MED number of high variability episodes was 10, and 100% of the CTG records met the requirements. Considering the low variability parameter, in normal CTG records, there should be no period of low variability. None of the low-variability episodes were observed in our series. The pathophysiological basis of the sinusoidal pattern is incompletely understood, but it often occurs in association with severe fetal anemia (i.e., in anti-D allo-immunization) [43]. No sinusoidal pattern was observed in our series; therefore, all the tracks were compliant with the requirement. Recording of fetal movement and recording of uterine contraction are parameters that do not affect the CTG record interpretation criteria, but give operators an additional key to interpreting the records [44].

Regarding fetal safety, secondary endpoints were considered a pre-term delivery and a five-minute APGAR score ≤ 7. No pre-term delivery had been observed, and the mean five-minute APGAR Score was 9.5 (2DS 0.5). No newborn had an APGAR score ≤ 7 at 5 min [45]. Therefore, considering fetal safety, even the two secondary endpoints were achieved in 100% of cases.

This study has some limitations: the first is the study design, which is retrospective and not prospective without any control group; the second is the relatively limited number of patients examined, and, moreover, the study is laking of a long-term neonatal follow-up. However, in our opinion, it also has some strengths, such as the high complexity of the cases examined and the completeness of the evaluation before the start of therapy with FCM. In particular, we would like to underline that the primary endpoint relating to fetal safety was assessed by recording the fetal heartbeat through the cardiotocographic tracing. To our knowledge, this is the first experience carried out using iron carboxymaltose. As a final consideration, we can point out that FCM was effective in treating anemia in this clinically complex population of pregnant women in the third trimester, and no safety concerns were identified in this study, although no definite conclusion about safety can be drawn from the results of this small case group. A prospective randomized controlled trial is warranted for a more detailed analysis of pregnancy outcomes.

## Figures and Tables

**Table 1:** Co morbidities from patients’ medical history.

Condition		Condition	
Diabetes	14 (13.3%)	Tuberculosis	2 (1.9%)
Hypotiroidism	12 (11.4%)	Pulmonary thromboembolism	1 (0.9%)
Hypertension	8 (7.6%)	Rheumatoid arthritis	1 (0.9%)
Multiple Abortions	4 (3.8%)	Severe asthma	1 (0.9%)
CNS disease	3 (2.9%)	Previous Fallot tetralogy	1 (0.9%)
Tyroiditis	3 (2.9%)	Sleeve gastrectomy	1 (0.9%)

Table 1 reports the comorbidities detected in the clinical history of the 105 patients considered in the study.

**Table 2:** Patients’ drug history.

Drug		Drug	
Oral iron	37 (35.2%)	Antibiotics	10 (6.5%)
Folates supplementation	35 (33.3%)	Antihypertensives	8 (7.6%)
B12 supplementation	31 (29.5)	Anti-inflammatories	8 (7.6%)
Insulin	14 (13.3%)	Tapazole	3 (2.9%)
Eutirox	12 (11.4%)	Corticosteroids	2 (1.9%)

Table 2 shows the pharmacological history relating to the therapies followed continuously.

**Table 3:** Symptoms.

Symptom	Results	Symptom	Results
Asthenia	87 (82.9%)	Fatigability	75 (71.4%)
Mucocutaneous pallor	52 (49.8%)	Hypotension	31 (29.5%)
Tachycardya	11 (10.5%)	Dyspnea	7 (6.7%)
Edema of the legs	7 (6.7%)	Irritability	1 (1.9%)

Table 3 reports the main symptoms reported by patients in their recent clinical history.

**Table 4:** Laboratory findings.

Parameter	Results	Parameter	Results
Hb before FCM (g/L)	MED 95 (IQR 8.5)	Vitamin B12 deficiency	N° 24 (22.8%)
MCV (fL)	MED 78 (IQR 13)	Folate deficiency	N° 14 (13.3%)
Ferritin (ng/mL)	MED 8 (IQR 11)	Hb disorders (total)	N° 18 (17.1%)
Transf Sat %	MED 10 (11 IQR)	B thalessemia Het	N° 13 (12.4%)
Iron μg/dL	MED 42 (IQR 58)	HbE	N° 3 (2.9%)
Hb after FCM (g/L)	MED 117 (IQR 1.5)	HbS	N° 2 (1.9%)
High CRP	N° 8 (7.6%)	Impaired EGFR	N° 3 (2.9%)
Hypophosphatemia	N° 6 (5.7%)		

Table 4 reports the most significant laboratory findings. Hb: hemoglobin, FCM: ferric carboxymaltose, MCV: mean corpuscular volume, Transf Sat: transferrin saturation. CRP: C-reactive protein, EGFR: estimated glomerular filtration rate, MED: median, IQR: interquartile range.

**Table 5:** Cardiotocography parameter in 128 procedures of sixty minutes.

Parameter	Expected Values	Observed Values MED (IQR)
Signal loss	<30%	4% (9%)
Basal heart rate	110–160 beats/min	137 (10) beats/min
Heart rate accelerations > 10 beats/min	≥1 episode	3 (1.8) episodes
Heart rate accelerations > 20 beats/min	≥1 episode	3 (2.0) episodes
Heart rate decelerations > 10 beats/min	<2 episodes	0 (1) episodes
Heart rate decelerations > 20 beats/min	<1 episode	0 (0) episodes
Short-term variations	>4 ms	12.9 (5.9) ms
High variability	≥1 episode	10 (4) episodes
Low variability	<1 in 50 min	0 (0) episodes
Sinusoidal pattern	Absent	0 (0) sinusoidal pattern
Fetal movements	Not Applicable	5 (11) movements
Uterine contractions	Not Applicable	2 (1) contractions

Table 5 reports the most significant cardiotocography parameters, all 128 CTG records were normal.

## Data Availability

The data presented in this study are available on request from the corresponding author.
